# Treatment of established TH2 cells with 4μ8c, an inhibitor of IRE1α, blocks IL-5 but not IL-4 secretion

**DOI:** 10.1186/s12865-018-0283-7

**Published:** 2019-01-10

**Authors:** Cody Poe, Cheyanne Youngblood, Karissa Hodge, Kyeorda Kemp

**Affiliations:** 1 Department of Natural Sciences, Northeastern State University, 3100 New Orleans Street, Broken Arrow, OK 74014 USA; 2 Department of Foundational Medical Studies, Oakland University William Beaumont School of Medicine, 586 Pioneer Drive, Rochester, MI 48309 USA

**Keywords:** TH2, IL-5, IRE1α, 4μ8c, Protein secretion

## Abstract

**Background:**

T cell activation induces ER stress and upregulates Inositol Requiring Enzyme 1 alpha (IRE1α), an activator of the unfolded protein response (UPR) pathway. Inhibition of IRE1α RNase activity in activated CD4^+^ splenocytes from naïve mice, via treatment of the cells with the commercially available drug 4μ8c upon activation, results in the reduction of the secretion of proteins IL-5, IL-4, and IL-13. Prior to this work, it was unknown if 4μ8c could inhibit TH2 cytokines in established TH2 cells, cells that are crucial in promoting disease in severe asthma.

**Results:**

Treatment of a mouse T helper (TH)2 cell line and differentiated human TH2 cells with 4μ8c resulted in inhibition of IL-5, but not IL-4, as measured by ELISA. The reduced cytokine expression was not due to differences in mRNA stability or mRNA levels; it appears to be due to a defect in secretion, as the cells produce cytokines IL-5 as measured by flow cytometry and western blot.

**Conclusion:**

These data suggest that the inhibition of IL-5 was due to post-translational processes. IL-5 promotes chronic, inflammatory asthma, and 4μ8c blocks its expression in T cells in vitro. Future studies will determine if 4μ8c treatment can ameliorate the effects of the cytokine IL-5 in a disease model.

**Electronic supplementary material:**

The online version of this article (10.1186/s12865-018-0283-7) contains supplementary material, which is available to authorized users.

## Background

Upon activation and differentiation, the endoplasmic reticulum (ER) of T cells is inundated with newly formed proteins that must be folded and exported to appropriate places in the cell. Failure of proteins to fold correctly leads to aggregates of misfolded proteins that induce stress in the ER. If this stress is not resolved, the cells die via apoptosis. To avoid apoptosis, cells have developed a response mechanism to this stressed state known as the unfolded protein response (UPR). The UPR is comprised of three conserved pathways that are named after the following initiating molecules: protein kinase RNA-like endoplasmic reticulum kinase (PERK), activating transcription factor 6 (ATF6), and inositol-requiring enzyme 1 alpha (IRE1α). PERK decreases general translation of proteins, while ATF6 and IRE1α increase the transcription of those that promote protein folding and degradation [1].

The UPR plays an integral part in the development and differentiation of T cells. ER stress and activation of the UPR is associated with altered T helper differentiation and cytokine secretion in patients with inflammatory diseases [2]. UPR inhibits IL-4/IL-13 signaling in T helper cells [3], and Eukaryotic Translation Initiation Factor 2α (EIF2α) regulates IL-4 transcription in primed TH2 cells [4]. Knocking out IRE1α halts the development of T cells at the CD4^−^CD8^−^ double-negative stage [5], and inhibition of IRE1α in primary mouse CD4 T cells undergoing activation using a commercially available drug, 4μ8c, results in decreased IL-4, IL-5, and IL-13 [6].

IL-4, IL-5, and IL-13, while important for promoting the clearance of parasites, can promote a disease state when improperly expressed, such as with asthma and allergy, by activating immune cells involved in these pathologies. Inhibition of TH2 cells and TH2 cytokines improves asthma and allergy outcomes in humans and animal models [7, 8]. This makes 4μ8c of potential interest for treatment of type 2 cytokine mediated diseases.

It is known that naïve T cells, cells undergoing differentiation, and T cells with an established phenotype have differences with regards to gene expression and regulation. Therefore, the results observed in naïve cells undergoing differentiation in the presence of 4μ8c are not necessarily representative of the effects of 4μ8c on established T cells. This work attempts to better understand the underlying mechanism of how inhibition of IRE1α by 4μ8c affects secretion of TH2 specific cytokines in established TH2 cells.

## Results

### IRE1α inhibition in established TH2 cells results in reduced IL-5, but not IL-4

**Fig. 1:**
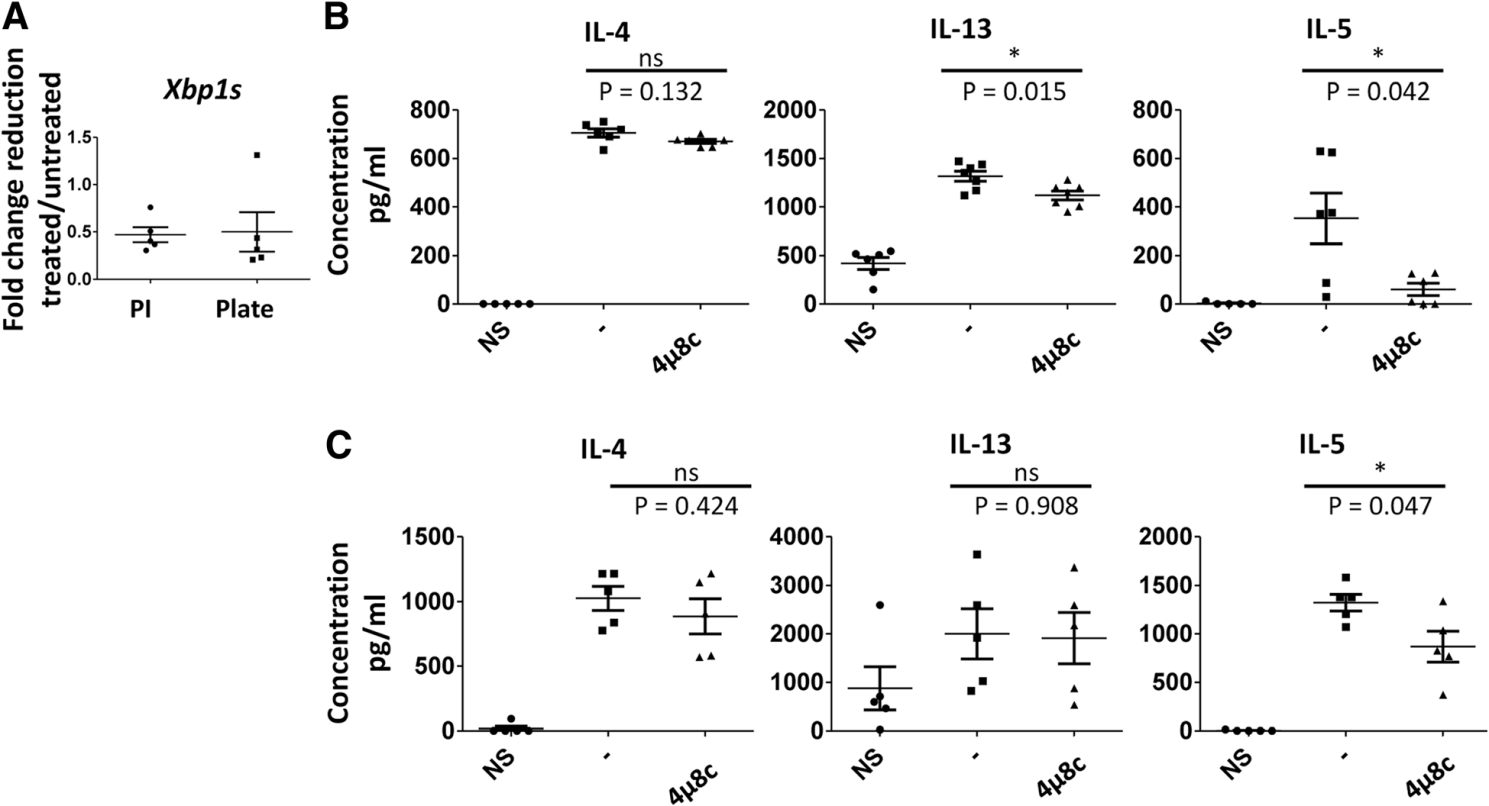
IL-5 is reduced in established mouse TH2 cells upon treatment with 4μ8c. D10 cells were rested in complete T cell media for 24 h at 37 °C. The cells were then left un-stimulated (NS) or stimulated with PMA and ionomycin (PI) or plate-bound α-CD3 and α-CD28 in the presence or absence (−) of 4μ8c for 24 h. **a** As a control the level of spliced *xbp1* mRNA was measured by qRT-PCR, as 4μ8c blocks the ability of IRE1α to cleave *xbp1*. The data shown is the fold change in reduction of treated vs. untreated after normalizing to the ns control for five experiments. The supernatants were harvested, and ELISA was performed from these samples as shown in B and C. **b** The data shown is from six experiments where cells were re-stimulated with PMA and ionomycin in the presence or absence (−) of 4μ8c. **c** The data shown is for five experiments where the cells were re-stimulated with plate-bound antibodies in the presence or absence (−) of 4μ8c. The standard error, upper and lower bars, and the mean, middle bar, is shown in all graphs. Hypothesis testing was done by Student’s T test unpaired, Welch’s correction (*p* value < 0.05)

IRE1α inhibition reduces cytokine secretion in primary T helper cells undergoing TH2 differentiation [6]. However, the cells that help to promote disease in many chronic disorders have an established phenotype. Therefore, it is important to develop treatments that are effective against these cells. In this study, we initially sought to determine how treatment of established TH2 cells with the commercially available small molecule inhibitor 4μ8c affects cytokine secretion. This inhibitor functions by binding to IRE1α and blocking its RNase activity, but not its kinase activity, resulting in reduced *X-box binding protein 1* (*xbp-1)* splicing [9]. The concentration of 4μ8c used in these experiments was determined by treating cells with varying concentrations of the inhibitor and then measuring cytokine secretion via ELISA and determining the number of cells that were alive after treatment (Additional file 1: Figure S1). In order to confirm that IRE1α was indeed inhibited, *xbp1s* was measured by qRT-PCR. It was reduced by around 50% in cells treated with 4μ8c (Fig. 1a). The murine TH2 cell line D10.G4.1 (referred to as D10) [10] was stimulated with phorbol 12-myristate 13-acetate (PMA) and ionomycin, strong agonists that activate molecules downstream of the T cell receptor (TCR) and CD28, in the absence (DMSO treated control cells) or presence of the IRE1α inhibitor 4μ8C. Then, IL-4, IL-13, and IL-5 protein expression was measured by ELISA. D10 cells that were treated with 4μ8c had reduced IL-5 and, to a lesser degree, IL-13 protein secretion compared to the control, while IL-4 levels appeared unchanged (Fig. 1b).

In order to validate that the results observed were not due to the stimulation protocol, the cells were stimulated with plate-bound antibody against CD3 and CD28. We found IL-5 to be significantly reduced, albeit to a lesser degree than in 1b, while IL-13 levels were similar to normal (Fig. 1c). This implied that the strength of signal in conjunction with 4μ8c could influence inhibition of IL-5 and IL-13. In order to confirm that treatment with 4μ8c did not affect cell viability, thereby resulting in reduced cytokine expression, we measured annexin V and propidium iodide (PI) staining and analyzed the number of live cells recovered after incubations. No difference was observed after stimulation with PMA and ionomycin (Additional file 1: Figure S1d and e).

**Fig. 2:**
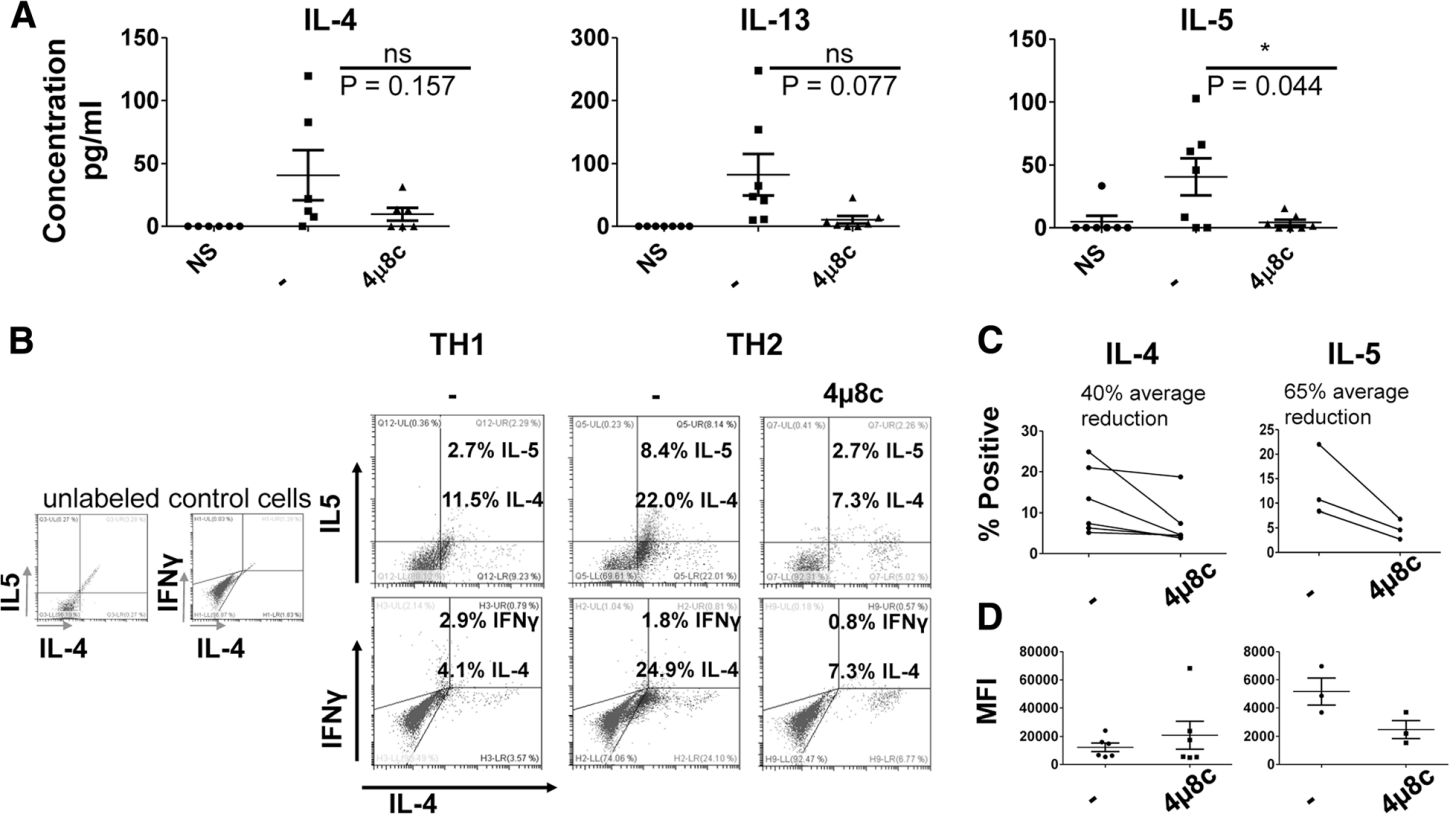
IL-5 is reduced in established human TH2 cells upon treatment with 4μ8c. **a** Blood was collected from seven individual volunteers in total. Cells were harvested from the blood using Ficoll, and CD4^+^ cells were isolated using Dynabeads. The cells were activated with plate-bound α-CD3 and α-CD28 for eleven days under TH2 conditions (IL-2, IL-4, α-IFNγ, and α-IL-12). The cells were rested for 24 h and then re-stimulated with plate-bound antibodies for 24 h in the presence or absence (−) of 4μ8c. An ELISA was performed on the supernatants. Of note, for TH2 cells differentiated from one individual, we were unable to detect IL-5 after differentiating the cells for 11 days, and that sample was removed from the IL-5 analysis, leaving us with an N of six. One of the samples from an individual was lost prior to running the ELISA for IL-4, leaving us with an N of six. The data shown are the results of six individual human samples for IL-4 and IL-5 and seven individual human samples for IL-13. The standard error, upper and lower bars, and the mean, middle bar, is shown in all graphs. Hypothesis testing was done by Student’s T test unpaired, Welch’s correction (*p* value < 0.05). **b** CD4^+^ cells were isolated from human blood as in A, activated under TH2 or TH1 conditions (IL-2, IL-12, and α-IL-4) for three days, and then stimulated with PMA and ionomycin in the presence of monensin for four hours. Intracellular staining was performed. The results are representative of six samples for IL-4 and three samples for IL-5. **c** The results for the percent positive and mean fluorescence intensity (MFI) **d** for IL-4 and IL-5 in treated and untreated cells differentiated three days in the presence of 4μ8c is shown for all intracellular flow experiments performed

Because the experiments above were performed using a cell line, we differentiated human cells for 11 days under TH1 and TH2 conditions. We then rested the cells for one day and stimulated the cells with plate-bound α-CD3 and α-CD28 for 24 h. We found that IL-5 was reduced in 4μ8c treated TH2 cells, while there was no statistically significant difference between 4μ8c treated and the untreated when IL-4 and IL-13 were measured (Fig. 2a). Moreover, as previously reported, cytokine IFNγ was not affected in cells cultured under TH1 conditions in the presence of 4μ8c, nor was IL-2 in TH1 and TH2 cells treated with 4μ8c (Additional file 2: Figure S2).

Treatment of mouse cells undergoing differentiation with 4μ8c inhibits IL-4 by 50% as measured by flow cytometry [6]. We found treatment of established TH2 cells resulted in loss of IL-5 secretion, but not IL-4. We postulated that this could be due to differences in gene regulation in a mouse system vs. a human system. Therefore, we differentiated human T cells under TH2 conditions in the presence of the inhibitor for three days. We found a trend towards reduced IL-4 and IL-5 producing cells upon differentiation in the presence of 4μ8c, 40 and 65% respectively (Fig. 2b-c). Interestingly, while the number of IL-4 producers decreased, the mean fluorescence intensity (MFI) was similar for IL-4 between treated and untreated cell populations. However, both the amount and percent of IL-5 producers appears to be reduced upon treatment with 4μ8c.

### Inhibition of IL-5 is due to post-transcriptional regulation

**Fig. 3:**
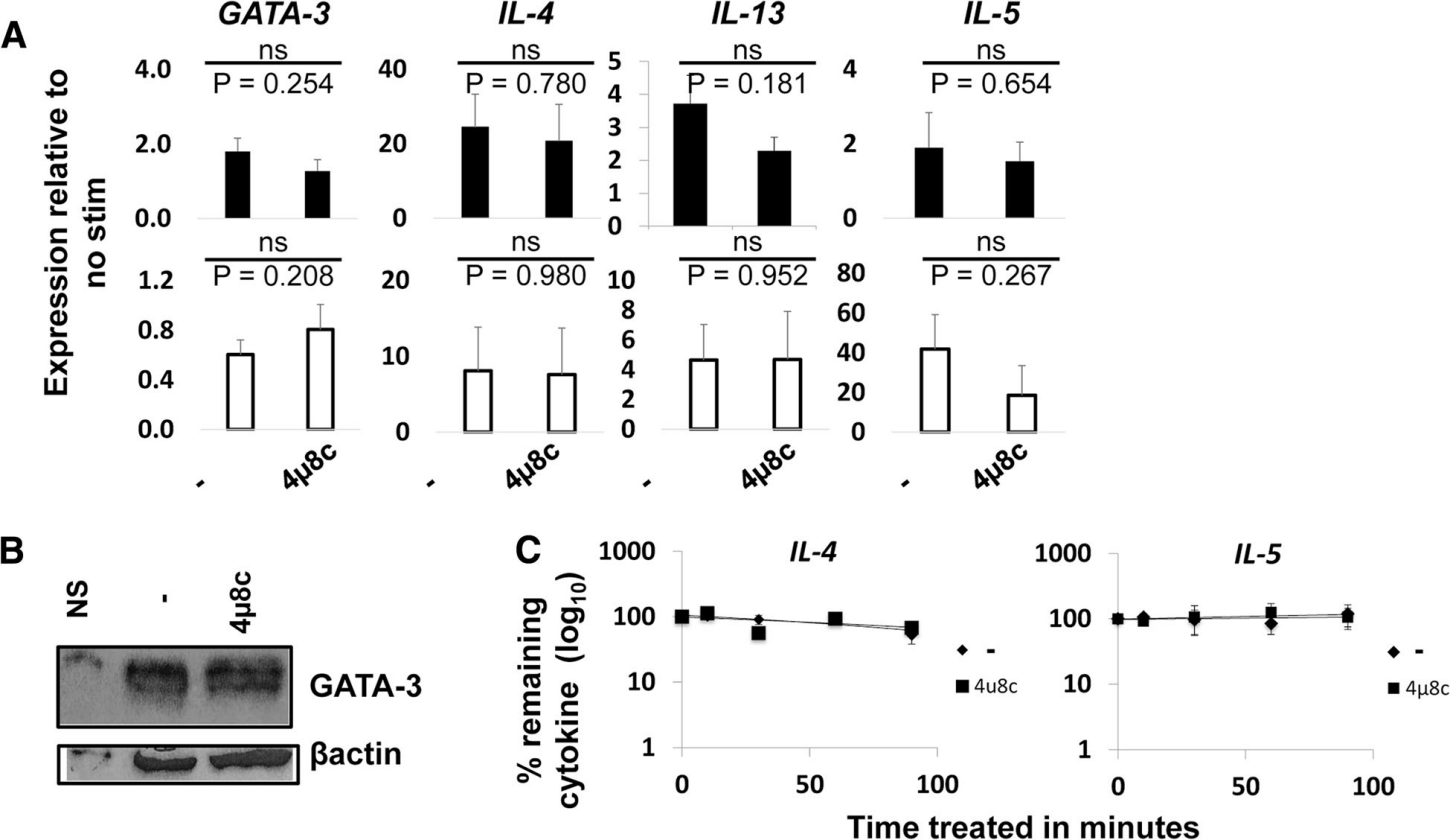
The reduction of IL-5 in 4μ8c treated cells is not due to changes in mRNA levels or stability. D10 cells were treated as in Fig. 1. **a** RNA was converted to cDNA and then amplified via qRT-PCR. The results show the relative fold change to the no stimulated sample. The data is an average of six experiments for the PMA and ionomycin stimulated samples (black bars) and five for the plate-bound stimulated ones (white bars). The standard error is graphed. **b** D10 cells were rested and then stimulated in the presence of 4μ8c for 24 h. The cells were then treated with actinomycin D and harvested at times 0, 10, 30, 60, and 90 min after treatment. RNA was isolated and qRT-PCR performed. The samples were normalized to the time zero point of actinomycin D treatment. The data were graphed on a semi-log scale and are the average of four experiments. The error bars represent the standard error of the mean. **c** Protein was isolated from cells treated as in A and immunoblotted with GATA-3 and β-actin antibody. The data is representative of three experiments

Previous studies show that loss of GATA-3 in established TH2 cells results in reduced IL-5 and IL-13, but not IL-4 [11]. Because treatment of D10 and established human TH2 cells resulted in reduced IL-5 secretion, but not IL-4, we measured GATA-3 expression in D10 cells stimulated in the presence or absence of 4μ8c. We found GATA-3 to be normal by qRT-PCR and western blot (Fig. 3a and b). In an effort to understand how 4μ8c influences IL-5 and IL-13 production, we also measured mRNA expression by qRT-PCR for IL-4, IL-5, and IL-13 in activated D10 cells, treated with 4μ8c or untreated. No significant reduction in mRNA levels of the cytokines tested was observed in cells treated with 4μ8c when compared to the untreated control (Fig. 3a).

CD4^+^ IRE1α deficient T cells differentiated under TH2 cells have reduced *IL-4* mRNA stability [12]. Therefore, we next investigated if IRE1α inhibition by 4μ8c negatively influenced cytokine mRNA stability in the established cell line. We focused our attention on IL-5 because of the dramatic reduction that occurred when established cells were treated with 4μ8c under all conditions tested. D10 cells were stimulated with PMA and ionomycin as above in the presence or absence of 4μ8c for 24 h, and then treated with actinomycin D to induce transcriptional arrest. The cells were harvested at time 0, 10, 30, 60, and 90 min after actinomycin D treatment. The mRNA was analyzed by qRT-PCR. There was no difference in the stability of *IL-4* or *IL-5* mRNAs in the established TH2 cells (Fig. 3c), leading us to conclude that inhibition of IL-5 by 4μ8c causes reduced IL-5 secretion through post-transcriptional mechanisms.

### IL-5 protein is made in established TH2 cells treated with 4μ8c, however it is not secreted

**Fig. 4:**
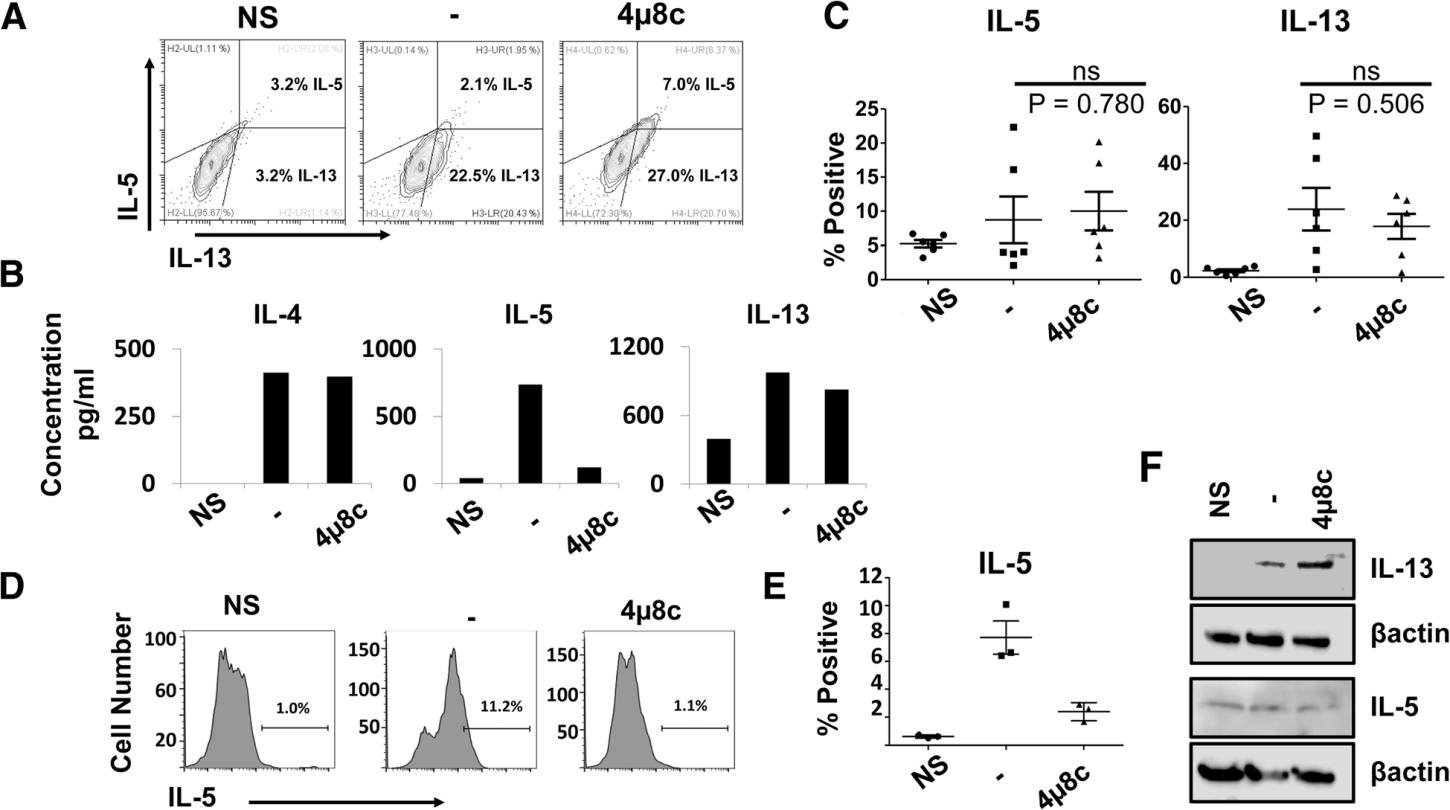
D10 cells treated with 4μ8c produce IL-5, but they cannot secrete it. D10 cells were treated as in Fig. 3a. **a** At 20 h, the cells were treated with monensin for an additional 4 hours and intracellular staining was performed for IL-5 and IL-13. **b** The supernatants were isolated at 20 h from the cells shown in A and an ELISA was performed in duplicate for IL-4, IL-5, and IL-13. The data is representative of six experiments. **c** The average percent of cells positive for IL-5 and IL-13 are graphed from six experiments. The error bars represent the standard error of the mean. Hypothesis testing was done by Student’s T test unpaired, Welch’s correction (p value < 0.05). **d** A cytokine secretion assay was performed on D10 cells treated as above. The data shown is a representative of the percent IL-5 secreting cells from each sample group. The experiment was done with duplicate samples three times. **e** The percent IL-5 secreting cells for each treatment that was performed in duplicate was averaged and graphed for the three individual experiments performed. **f** Protein was isolated from cells treated as in 3A and immunoblotted with IL-5, IL-13, and β-actin antibody. The data is representative of two experiments

PERK, a member of the UPR that promotes translational arrest, regulates translation of the type 2 cytokine IL-4 in primed TH2 cells in response to TCR stimulation [4]; therefore, we hypothesized that the observed cytokine deficiency upon 4μ8c treatment could be due to a reduction in the production or the secretion of IL-5. We measured IL-5 and IL-13 via flow cytometry, cytokine secretion assay, ELISA, and western blot (Fig. 4) in D10 cells stimulated with PMA and ionomycin and treated with 4μ8c as in Fig. 1. We found IL-5 and IL-13 were made in D10 cells treated with 4μ8c, as indicated by flow cytometry and western blot (Fig. 4a, c, and f). However, there was a reduction in IL-5 secretion as measured by ELISA and cytokine secretion assay (Figs. 1 and 4b, d, and e). While these cells made IL-5, they displayed reduced expression of IL-5 in their supernatants (Figs. 1 and 4a) and had decreased IL5 cytokine secretion (Fig. 4c), indicating that the defect in IL-5 was due to a failure to secrete this protein.

## Discussion

### Why is IL-4 secretion inhibited in cells undergoing differentiation when treated with 4μ8c, but not in established cells?

Naïve and established T cells differ in gene expression and regulation. Indeed, transcription factor GATA-3 is required for establishing IL-4, IL-5, and IL-13 expression in naïve T cells undergoing TH2 differentiation, but is not required for IL-4 expression in established TH2 cells [11]. Our data and a previous study suggest that while IL-4, IL-5, and IL-13 are inhibited in cells undergoing differentiation when treated with 4μ8c, IL-5, but not IL-4, is inhibited in established cells (Figs. 1 and 2 and [6]). This reduced cytokine expression appears to be due to changes in post-translational regulation, as the protein is detected inside of the cell, but not secreted. Interestingly, we found differentiation of human T cells in the presence of 4μ8c reduced the percent of IL-4 producers, but the mean fluorescence intensity (MFI) was similar between 4μ8c untreated and treated groups. This indicates that the small subset of cells that retain the ability to make IL-4, have increased IL-4 production. We are unsure why this phenomenon is occurring.

Kemp et al. [12] found T cells from IRE1α mutant mice had reduced p38 activation, and the p38 MAP kinase pathway is implicated in post-translational regulation [13, 14]. Moreover, the p38 MAP kinase pathway plays disparate roles in TH2 cytokine expression in naïve cells undergoing differentiation vs. memory cells [15–17]. The inhibition of p38 in human CD4^+^ T cells results in reduced IL-4, IL-5, and IL-13 [15, 17], while inhibition of p38 in established human cells partially inhibited TH2 cytokines [15]. Mori et al. found inhibition of p38 in established human TH2 clones from atopic asthmatic patients resulted in reduction of IL-5, but not IL-4, IL-2, or IFNγ [16]. Cytokine IL-33 further enhances IL-5 and IL-13 production in established human TH2 cells [18, 19], and this is dependent on signaling via p38 MAP kinase [18]. Recently, 4μ8c was shown to suppress IgE mediated activation of mast cells via inhibition of the p38 MAP kinase pathway [20]. Future studies will have to determine if 4μ8c regulates IL-5 and IL-13 post-translationally via the p38 MAP kinase pathway in established TH2 cells.

### Why is IL-5 made, but secretion is inhibited by treatment with 4μ8c?

Cytokine IL-5 is made in TH2 cells treated with 4μ8c, but not secreted. This could be due to decreased protein stability, reduced vesicular trafficking, or a combination of both. Because we can detect normal levels of IL-5 in the cell via western blot and flow cytometry, we expect that vesicular trafficking is being hindered in some manner. The IRE1α pathway has been implicated in the regulation of vesicular trafficking [21–25], and it is possible that IL-5 fails to travel to the plasma membrane in cells treated with 4μ8c. Interestingly, p38, which 4μ8c inhibits in mast cells, is implicated in regulating vesicular trafficking [26].

Cytokine vesicular trafficking in immune cells is not clearly defined or understood. Regulation is dependent on cell type and is situational. We hypothesize that treatment with 4μ8c inhibits IL-5 vesicular trafficking. Future studies will determine how vesicular trafficking of type 2 cytokines is regulated in T cells, and whether treatment of established TH2 cells with 4μ8c results in deficient vesicular trafficking.

4μ8c inhibits the RNase domain of IRE1α, which blocks activation of UPR via XBP-1 [9]. While this study demonstrates that 4μ8c inhibits IL-5 in established TH2 cells, but not IL-4, we did not show that this occurred directly due to inhibition of IRE1α. It is possible that the results were due to off-target effects. However, previous studies using 4μ8c show the drug to act specifically on the IRE1α/XBP-1 pathway [6, 27, 28]. In addition, we did not find 4μ8c treatment to effect type 1 cytokines or apoptosis (Fig. 2, Additional files 1 and 2: Figures S1 and S2, and [12]).

### 4μ8c and disease treatment

Hypersensitivity reactions involving the immune system, commonly known as allergies or allergic diseases, are a common problem in  high income earning nations. Type I hypersensitivities are linked to excessive IgE production and type 2 cytokines. Upon encounter with the allergen during the sensitization phase, CD4^+^ cells proliferate and acquire the ability to produce type 2 cytokines. These cytokines direct many activities in the body: IL-4 and IL-13 promote antibody isotype switching; IL-5 and IL-13 induce eosinophil differentiation and maturation; and IL-13 promotes airway hyper-responsivity, upregulation of macrophages and increases mucus in the airway [7, 29].

Recently, a subset of memory TH2 cells found to express IL-5, IL-4, and IL-13, termed tpath2 cells, have been highlighted in inducing allergy and asthma. They play a crucial role in inflammatory disorders, such as asthma, and have been described in human and mouse models of allergy [30]. The majority of patients with asthma are able to control symptoms with current drug regimens; however, a subset of patients have severe asthma, and increased morbidity, mortality, and treatment costs are associated with this group [31]. The majority of patients with severe adult onset asthma can be characterized as having eosinophilic asthma [32, 33]. Moreover, increased eosinophilic inflammation is found in children with severe asthma [34]. Treatment with drugs that inhibit IL-5 improve quality of life and asthma symptoms [35–37]. Many of these drugs target the IL-5 receptor or IL-5 cytokine; however, 4μ8c appears to target IL-5 secretion, and this makes 4μ8c of interest for the treatment of asthma due to the ability of 4μ8c to target IL-5 in established TH2 cells.

4μ8c may also prove to be effective against other cells implicated in asthma and allergy. Innate lymphoid type 2 cells share many functional similarities with tpath2 cells and conventional TH2 cells [38] and are implicated in allergy and asthma [39–41]. Moreover, 4μ8c was recently found to decrease passive cutaneous anaphylaxis in mice, a syndrome in which mast cells play a major role [20]. These data point to the potential of 4μ8c to target a variety of cells in hypersensitivity disorders.

## Conclusion

In summary, our results indicate that 4μ8c inhibits the secretion of IL-5 in established TH2 cells, but not IL-4. This is of importance because established effector cells contribute greatly to disease in chronic inflammatory disorders. This work and other recent studies suggest a role for 4μ8c as a candidate for the treatment of allergy and asthma.

## Materials and methods

### Aim and design of the study

The aim of this study was to determine how treatment of established TH2 cells with 4μ8c effected TH2 cytokine expression. D10.G4.1 (mouse TH2 cell line) and human TH2 cells were treated with 4μ8c and downstream applications, as explained below, were performed. All work was performed at Northeastern State University.

### Human subjects

Blood was collected from human volunteers of both sexes between the ages of 18–65 by a trained phlebotomist. All volunteers self-reported as being healthy.

### Ethics, consent, and permission regarding human subjects

All human subjects read and signed a consent form after being presented with an opportunity to ask questions related to the study. All subjects were informed of their right to request removal from the study. This study was conducted as approved by the Institutional Review Board at Northeastern State University (In vitro studies of ER gene expression upon T cell activation -IRB # 17–058).

### Culture of established TH2 murine cell line D10.G4.1

D10.G41 (D10) cells are a TH2 T cell clone derived from AKR/J mice. Their TCR recognizes conalbumin peptide CA 134–146 in the context of IA^k^ [10]. These cells were gifted to our lab by Dr. Deyu Fang (Northwestern University), but they were originally obtained from the American Type Cell Culture Collection (ATCC; Manassas, Va) and cultured based on the recommendations of ATCC. Briefly, the cells were cultured at 37 °C with 5% CO_2_ in RPMI complete T cell media (RPMI-1640 + L-glutamine, 10% FBS, 50 μM 2-mercaptoethanol, 10 mM HEPES, 1 mM sodium pyruvate, and penicillin/ streptomycin) at a concentration of 2 × 10^5^ cells/mL and suspended in fresh media every two to three days. The cells were treated with IL-2 (10 ng/mL), IL-1α (10 pg/mL), and conconavalin A (2 μg/mL) to induce growth. The media and supplements were obtained from Invitrogen, the cytokines were obtained from PeproTech Inc., and the conconavalin A was obtained from Sigma Aldrich.

### CD4^+^ T cell purification and in vitro differentiation of TH cells

Blood was collected from seven individual volunteers in total. We sent out a request for blood three different times. Two individuals volunteered two of the times, and on one occasion, three individuals volunteered, giving us a total of 7 individual participants.

Peripheral mononuclear cells were isolated from human blood using Ficoll (Millipore) according to the manufacture’s guidelines. CD4^+^ T cells were positively selected using the Dynabead isolation kit (Life Technologies). Purified CD4^+^ cells were plated in 96 well (0.1 × 10^6^/well) or 24 well dishes (0.5 × 10^6^/well) that were coated with 5 μg/ml of α-CD28 (OKTϵ) and 2 μg/ml of α-CD3 (145-2c11) and cultured under TH1 (10 ng/ml IL-2, 10 ng/ml IL-12 and 1 μg/ml α-IL-4 [8D4–8]) or TH2 (10 ng/ml IL-2, 20 ng/ml IL-4, 1 μg/ml α-IL-12 [C8.6], and 1 μg/ml α-IFN-γ [NIB42]) skewing conditions in RPMI complete T cell media for three or eleven days. For cells cultured eleven days, the cells were split into new wells that were coated with antibody every two to three days. On day seven of the eleven day culture, the cells were harvested, counted, and plated with fresh media. The cells were then maintained as before. All cytokines were purchased from PeproTech Inc., and all antibodies were purchased from Biolegend.

### 4μ8c treatment of D10 cells

The cells were suspended in complete T cell media at a concentration of 0.5 × 10^6^/mL or 1 × 10^6^/mL for 24 h at 37 °C with 5% CO_2_ in the absence of stimulation. The cells were then harvested and transferred to culture treated plates at a concentration of 1 × 10^6^/mL in complete T cell media under no stimulation (NS) or stimulation consisting of ionomycin (1 μM) and phorbol 12-myristate 13-acetate (PMA) (25 ng/mL) or plate-bound α-CD3 (2 μg/ml) and α-CD28 (5 μg/ml) in the presence of 4μ8c (10 μg/mL) or equal volume of dimethyl sulfoxide (DMSO). DMSO, PMA, and ionomycin were obtained from Sigma Aldrich. 4μ8c was obtained from Millipore.

### 4μ8c treatment of human cells

CD4^+^ cells were differentiated under TH2 conditions as above for three days in the presence of 4μ8c (5 μg/ml) or equal volume of DMSO. In some experiments the cells were differentiated for eleven days, rested for one day, and then stimulated with plate-bound α-CD3 (145-2C11) and α-CD28 (clone 2.43, rat IgG) or PMA (25 ng/mL) and ionomycin (1 μM) for 20–24 h in the presence of 4μ8c or equal volume of DMSO as above.

### Analysis of cytokine expression by ELISA and flow cytometry

The cell supernatants were harvested from plates after stimulation and treatment with 4μ8c as stated above. ELISAs were conducted following the manufacture’s protocols for IL-2, IFNγ, IL-4, IL-5, and IL-13, and all ELISA kits were obtained from Biolegend, with the exception of the IL-13 kit. The IL-13 kit was obtained from Invitrogen. For flow cytometry experiments, D10 cells were treated as explained above with PMA and ionomycin or plate-bound α-CD3 and α-CD28 for 20 h when monensin (Biolegend) was added. The samples were incubated an additional four hours at 37 °C with 5% CO_2_. The cells were fixed and permeabilized using cytofix fixation buffer (Biolegend) according to the manufacturer’s instructions. Human T cells differentiated under TH2 conditions were stimulated as indicated above for three days in the presence or absence of 4μ8c, harvested, and stimulated with PMA and ionomycin in the presence of monensin for four hours as described above. The cells were suspended in fluorochrome-conjugated antibodies specific for mouse/human IL-5 (TRFK5, Biolegend) and IL-13 (abcam, AB95576) or human IL-4 (Biolegend, 8D4–8), and IFN-γ (Biolegend, 4S.B3) using the concentrations suggested by the manufacturer for 30 min at room temperature, washed, resuspended in FACS Buffer (2% BSA in 1x PBS), filtered, and analyzed via a Cytoflex flow cytometer (Beckman Coulter). IL-5 cytokine secretion assay was performed according to manufacture instructions (Miltenyi Biotec), with the exception that bovine serum was used in place of human serum and 80 μL 1x PBS was used in place of 80 μL cold buffer when adding the IL-5 Detection Antibody (PE). All flow cytometry data was analyzed using the Cytoflex or FLowJo v10 software.

### Cell viability assays

D10 cells were treated as explained above with PMA and ionomycin in the presence or absence (DMSO alone) of 4μ8c as indicated in the figure legend. Annexin V and PI staining was done on cells incubated in the presence or absence of 4μ8c as above according to the manufacturer guidelines (Biolegend). The cells that were left unstimulated or stimulated in the presence of absence of 4μ8c were incubated with 0.5 mg/ml of MTT (3-(4, 5-dimethylthiazolyl-2)-2, 5-diphenyltetrazolium bromide) reagent (Sigma-Aldrich) in a 96 well round bottom plate for two hours, washed with 1x PBS, and then lysed by incubating cells for 15 min at room temperature with 75% DMSO solution made in 1x PBS. The wells were immediately read by measuring absorbance at 595 nm wavelength on a Biomark plate reader (Biorad). In some experiments, the number of cells alive and dead were determined by counting cells with trypan blue (Sigma Aldrich) and calculating the percent alive of total cells.

### RNA isolation and qRT-PCR

RNA was isolated from cells using Trizol (Invitrogen) and reverse transcribed using the SuperScript IV Reverse Transcriptase kit (Invitrogen) or QScript cDNA mastermix (Quanta BioSciences) according to the manufacture’s guidelines. cDNA reactions were carried out using a MiniCycler (MJ Research).

qPCR was carried out using Power Sybr Green (Invitrogen) on a MiniOpticon Real-Time PCR System (Bio-Rad), and the relative expression was calculated as previously described [12]. β-actin was used to normalize all samples. Sample expression was then determined relative to the no stimulation control. The primers used in this study have been reported previously [6, 42].

### Western blot

Cells treated as above were lysed in RIPA buffer as previously described [12]. The lysates were run on a 4–20% gradient SDS gel (Biorad), transferred to nitrocellulose, blocked in 3% (for the GATA-3 blots) or 5% milk in TBST, and blotted with the antibodies against the following: β-actin (Thermofisher, MA515739), IL-5 (My biosource, MBS821891), IL-13 (Abcam, ab106732), and GATA-3 (Santa Cruz, 1A12-D9).

### Actinomycin D experiments

The cells were treated with 4μ8c or left untreated and stimulated with PMA and ionomycin as above for 24 h. The cells were then treated with actinomycin D (Sigma Aldrich) at a concentration of 3 μg/ml at 37 °C. The samples were harvested at 0, 10, 30, 60, and 90 min after exposure to actinomycin D. mRNA from all samples was isolated using Trizol, converted to cDNA, and analyzed via qRT-PCR as described above.

### Statistical analysis

The data was analyzed using a 2-tailed Student’s unpaired T test, Welch’s correction. Use of the test was based on the fact that the samples to be analyzed were independently related and the variances between data sets could not be assumed to be equal. In Additional file 2: Figure S2a, a one-way ANOVA was run. The test was used to determine if cytokine gene expression differed between independent sample sets. The samples were considered to differ significantly if the *p* value was less than 0.05. In all graphs the standard error is represented by error bars unless otherwise indicated. Power analysis was performed to determine the minimum number of samples to obtain for the experiments using previously published data where T cells were treated with 4μ8c and IL-4 was measured [6]. The study group design is for two independent groups, continuous data, α = 0.05, and power 80%, with the minimum number needed equal to three samples per group. All statistical testing and analysis was performed after the predetermined sample size was obtained for each experiment.

## Additional files

Additional file 1: **Figure S1**: Optimization for 4μ8c treatment. **(A)** D10 cells were rested in complete T cell media for 24 h at 37 °C. The cells were then stimulated with PMA and ionomycin for an additional 24 h in the presence of 4μ8c (+ is equal to 2.5 μg/ml, ++ is equal to 15 μg/ml, or +++ is equal to 45 μg/ml). As a control cells were treated with equal volumes of DMSO, as 4μ8c is resuspended in DMSO. Supernatants were harvested at 24 h and an ELISA was performed for IL-4 and IL-5. The data shown is representative of three experiments. **(B)** An MTT cell viability assay was performed on cells treated as in A. The absorbance was read and the data is graphed for the DMSO and 4μ8c treated cells relative to the stimulated sample control. The data is an average of two separate experiments. The standard deviation is shown. **(C-D)** D10 cells were rested and then stimulated as in A in the presence of increasing amounts of 4μ8c (10 μg/ml, 15 μg/ml, 25 μg/ml, 35 μg/ml, and 45 μg/ml). The supernatant was harvested at 24 h and an ELISA was performed for IL-5 **(C)**. The cells were harvested at 24 h and counted using trypan blue. The total number of cells and the live cells present were counted, and the percent live cells is graphed **(D)**. The data in C and D are representative of two experiments. **(E)** D10 cells were rested in complete T cell media for 24 h at 37 °C. The cells were then left un-stimulated (NS) or stimulated with PMA and ionomycin for an additional 24 h in the presence or absence of 4μ8c. The cells were then harvested and annexin V and PI staining was performed according to the manufacture’s guidelines. **(F)** The cell counts of D10 cells harvested from six individual experiments treated as in A are averaged and graphed. The standard error is graphed. (TIF 196 kb)

Additional file 2: **Figure S2**: Human cells treated with 4μ8c secrete IL-2 and IFNγ. The cells were harvested from human blood using Ficoll, and CD4^+^ cells were isolated using Dynabeads. The cells were activated with plate-bound α-CD3 and α-CD28 for 11 days under TH1 and TH2 conditions. The cells were rested for 24 h and then re-stimulated with plate-bound antibodies or 50 ng/ml of PMA and 1 μM ionomycin for 24 h in the presence or absence (−) of 4μ8c. An ELISA was performed on the supernatants. **(A)** The results from five (TH1- columns one and two) and six (TH2- columns three and four) samples are graphed for IL-2. The mean and standard error is shown. There is no statistically significant difference regarding IL-2 production for the TH1 and TH2 samples treated and untreated- 1way ANOVA [(F (3,18) = 1.096, *p* = 0.984)]. **(B)** The results from six samples are graphed for the IFNγ data. There is no statistically significant difference regarding IFNγ expression after treatment in TH1 cells, Student’s T test unpaired, Welch’s correction (*p* = 0.688). (TIF 74 kb)

## Abbreviations

ATF6: Activating transcription factor 6
D10: D10.G4.1
EIF2α: Eukaryotic Translation Initiation Factor 2α
ER: Endoplasmic reticulum
IRE1α: Inositol Requiring Enzyme 1 alpha
MTT: 5-diphenyltetrazolium bromide
NS: No stimulation
PERK: Protein kinase RNA-like endoplasmic reticulum kinase
PI: Propidium iodide
PMA: Phorbol 12-myristate 13-acetate
TCR: T cell receptor
TH: T helper
UPR: Unfolded protein response
XBP-1: *X-box binding protein 1*, and 3-(4, 5-dimethylthiazolyl-2)-2
