# Supplementary figures and images for: Treatment of established TH2 cells with 4μ8c, an inhibitor of IRE1α, blocks IL-5 but not IL-4 secretion
Source: BMC Immunol. 2019 Jan 10;20:3. doi: 10.1186/s12865-018-0283-7 (PMC6327572; doi:10.1186/s12865-018-0283-7)

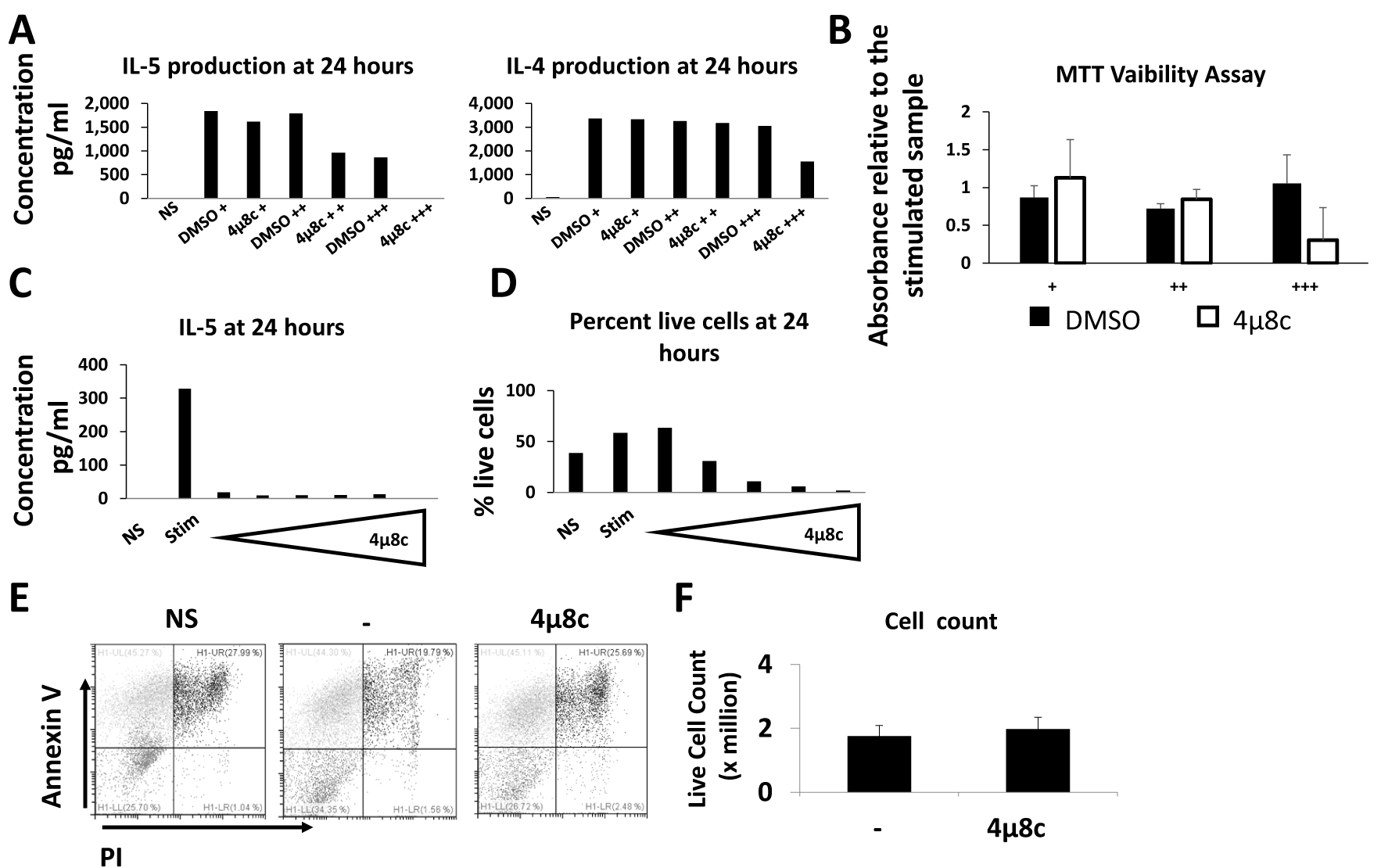

Supplement: Supplementary file 1 — Figure S1: Optimization for 4μ8c treatment. (A) D10 cells were rested in complete T cell media for 24 h at 37 °C. The cells were then stimulated with PMA and ionomycin for an additional 24 h in the presence of 4μ8c (+ is equal to 2.5 μg/ml, ++ is equal to 15 μg/ml, or +++ is equal to 45 μg/ml). As a control cells were treated with equal volumes of DMSO, as 4μ8c is resuspended in DMSO. Supernatants were harvested at 24 h and an ELISA was performed for IL-4 and IL-5. The data shown is representative of three experiments. (B) An MTT cell viability assay was performed on cells treated as in A. The absorbance was read and the data is graphed for the DMSO and 4μ8c treated cells relative to the stimulated sample control. The data is an average of two separate experiments. The standard deviation is shown. (C-D) D10 cells were rested and then stimulated as in A in the presence of increasing amounts of 4μ8c (10 μg/ml, 15 μg/ml, 25 μg/ml, 35 μg/ml, and 45 μg/ml). The supernatant was harvested at 24 h and an ELISA was performed for IL-5 (C). The cells were harvested at 24 h and counted using trypan blue. The total number of cells and the live cells present were counted, and the percent live cells is graphed (D). The data in C and D are representative of two experiments. (E) D10 cells were rested in complete T cell media for 24 h at 37 °C. The cells were then left un-stimulated (NS) or stimulated with PMA and ionomycin for an additional 24 h in the presence or absence of 4μ8c. The cells were then harvested and annexin V and PI staining was performed according to the manufacture’s guidelines. (F) The cell counts of D10 cells harvested from six individual experiments treated as in A are averaged and graphed. The standard error is graphed. (TIF 196 kb) [file 12865_2018_283_MOESM1_ESM.tif]

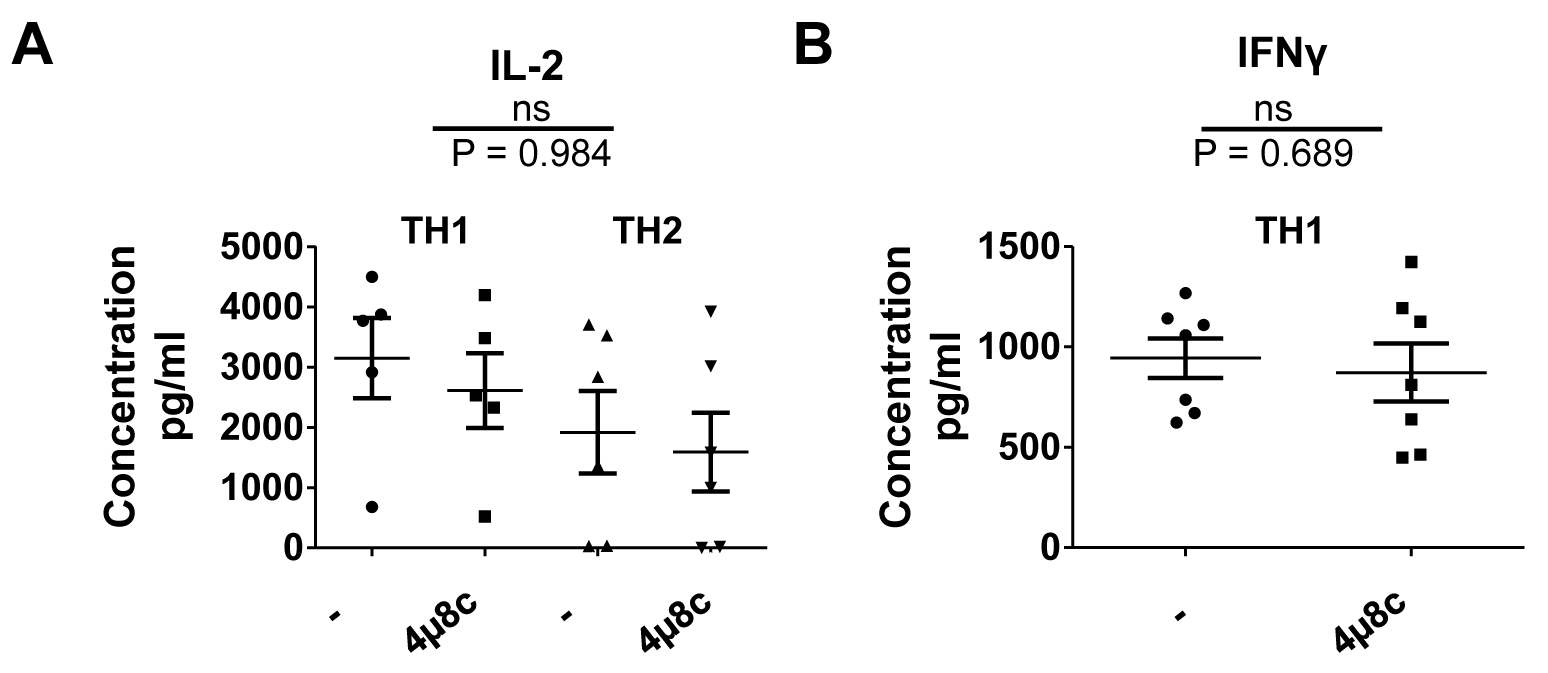

Supplement: Supplementary file 2 — Figure S2: Human cells treated with 4μ8c secrete IL-2 and IFNγ. The cells were harvested from human blood using Ficoll, and CD4+ cells were isolated using Dynabeads. The cells were activated with plate-bound α-CD3 and α-CD28 for 11 days under TH1 and TH2 conditions. The cells were rested for 24 h and then re-stimulated with plate-bound antibodies or 50 ng/ml of PMA and 1 μM ionomycin for 24 h in the presence or absence (−) of 4μ8c. An ELISA was performed on the supernatants. (A) The results from five (TH1- columns one and two) and six (TH2- columns three and four) samples are graphed for IL-2. The mean and standard error is shown. There is no statistically significant difference regarding IL-2 production for the TH1 and TH2 samples treated and untreated- 1way ANOVA [(F (3,18) = 1.096, p = 0.984)]. (B) The results from six samples are graphed for the IFNγ data. There is no statistically significant difference regarding IFNγ expression after treatment in TH1 cells, Student’s T test unpaired, Welch’s correction (p = 0.688). (TIF 74 kb) [file 12865_2018_283_MOESM2_ESM.tif]
